# Molecular Diversity and Isoform Evolution in *Tityus obscurus* Venom: Insights from Proteomic Analysis

**DOI:** 10.3390/toxins17050210

**Published:** 2025-04-23

**Authors:** Kemellyn Cristina Panchera, Lais Campelo Mendes, Ana Leonor Abrahão Nencioni, Daniel Carvalho Pimenta, Emídio Beraldo-Neto

**Affiliations:** 1 Programa de Pós-Graduação em Ciências—Toxinologia do Instituto Butantan, São Paulo 05503-900, Brazil; kpanchera@gmail.com (K.C.P.); lais.campelo21@gmail.com (L.C.M.); 2 Laboratório de Bioquímica do Instituto Butantan, São Paulo 05503-900, Brazil; dcpimenta@butantan.gov.br; 3 Laboratório de Farmacologia do Instituto Butantan, São Paulo 05503-900, Brazil; ana.nencioni@butantan.gov.br

**Keywords:** neurotoxins, scorpion toxins, sodium channel, potassium channel

## Abstract

Over millions of years of evolution, natural selection has driven the specialization of predatory and defensive mechanisms in various animal species through the development of poisons and venoms. These venoms contain highly specific and selective molecules for several different targets according to the habitat and behavior of each species. In this work, we performed a comprehensive proteomic analysis of *Tityus obscurus* venom, identifying 45 proteins, including 8 toxins targeting K^+^ channels, 22 targeting Na^+^ channels, and 15 other venom components. Our study reveals seven novel isoforms of ion channel-targeting peptides characterized by amino acid substitutions which may influence their bioactivity and pharmacological properties. These findings contribute to our understanding of venom molecular diversity and evolution, providing new insights into peptide structure–function relationships. Considering the biomedical relevance of ion channel-modulating toxins, our work expands the repertoire of potential candidates for future drug development, particularly in the context of neuropharmacology and ion channel disorders.

## 1. Introduction

Scorpions comprise a diverse group, with over 2200 species identified worldwide. Among them, more than 30 belong to the Buthidae family, but only 25 species are considered to be medically relevant. In Brazil, approximately 160 species have been recorded [1,2].

The species *Tityus obscurus* (also referred to as *Tityus paraenses* and/or *Tityus cambridgei*), commonly referred to as “lacrau” among rural populations and as the “black Amazon scorpion”, is predominantly found in tropical forests [3,4]. Its distribution includes countries such as French Guiana, Suriname, and Brazil, particularly in the northern states [4]. In the state of Pará, envenomations by this species represent the second most common cause of accidents involving venomous animals [3]

The neurotoxic effects observed in scorpion envenomation are primarily due to peptides, also referred to as neurotoxins, that act on ion channels [5,6]. Scorpion venoms comprise a diverse array of molecules, including free nucleotides, biogenic amines, lipids, proteins, and peptides, all of which play crucial roles in prey capture and defense against predators. Among the components of the venom, peptides have attracted considerable attention due to their significant biological activities [7]. The first recorded case of *Tityus obscurus* envenomation in the Amazon region was reported in French Guiana in 1950 [4]. Envenomation by *Tityus obscurus* can cause both local and systemic symptoms, primarily affecting the nervous system [8]. Symptoms include pain, erythema, tachycardia, sweating, and sialorrhea, along with neurological effects such as ataxia and dysmetria, among others [3]. More specifically, cases reported in the state of Pará have exhibited symptoms such as dysarthria (difficulty in articulating words) and myoclonus (involuntary muscle contractions) [4].

Previous transcriptomic studies on *Tityus obscurus* venom revealed that 11.4% of the venom’s transcripts encode toxins, particularly those targeting sodium and potassium channels, as well as metalloproteinases [3]. Batista et al. [9] employed high-performance liquid chromatography (HPLC) and mass spectrometry (ESI-MS and MALDI-TOF/MS) to partially identify 26 toxins and to fully sequence a toxin, Tc48a, from *Tityus obscurus* (then referred to as *Tityus cambridgei*). Later, Dias et al. [10] further explored the venom’s composition, identifying 360 peptides ranging from 400 to 4000 Da—27 of which were sequenced—and synthesized and functionally characterized 30 small linear peptides.

The peptides that compose the venom of these animals can be categorized into two groups: peptides with disulfide bonds (DBPs) and peptides without disulfide bonds (NDBPs). Peptides in the NDBP group exhibit various biological activities, including antimicrobial and immunomodulatory effects [11,12]. In contrast, DBP-group peptides are the primary molecules responsible for the neurotoxic effects characteristic of scorpion envenomation, as they modulate cellular ion channels [7,13,14].

Peptides targeting Na^+^ and K^+^ ion channels play distinct yet critical roles in the neurotoxic effects associated with scorpion envenomation. Peptides that act on Na^+^ ion channels typically consist of approximately 55–76 amino acid residues linked by three or four disulfide bonds [15,16]. These peptides are considered the primary contributors to neurotoxicity in Brazilian scorpions. In contrast, peptides interacting with K^+^ and Cl^−^ channels are generally smaller, comprising around 30 to 40 amino acid residues [7,17].

Toxins targeting K^+^ ion channels disrupt various physiological processes by modulating neuronal activity, neurotransmitter release, hormone secretion, lymphocyte activation, muscle contraction, and the cardiac cycle. Neurological disturbances resulting from these interactions are particularly associated with the symptoms of scorpion envenomation. Additionally, some of these peptides have evolved to act selectively on ion channels of either mammals or insects, enhancing their specificity and potency [18,19].

Meanwhile, toxins targeting Na^+^ ion channels are classified into α and β types, each with distinct mechanisms of action. α-Toxins specifically inactivate Na^+^ channels by inducing prolonged states of cell depolarization. By binding to site 3 of these channels, α-toxins slow the inactivation of sodium currents and prolong the action potential. These toxins are further divided into three categories: classical α-toxins (highly active in mammalian brains), insect α-toxins, and α-like toxins (active in the central nervous systems of both mammals and insects) [20,21]. On the other hand, β-type toxins affect Na^+^ channel activation by causing changes that lead to cell hyperpolarization [20]. As highlighted by [21], β-toxins bind to site 4 receptors on Na^+^ channels, shifting the activation voltage to more negative membrane potentials and reducing peak amplitude. Notably, β-toxins demonstrate selectivity towards specific isoforms of sodium channels present in both mammals and insects [21].

Given the importance of these peptides, the discovery and characterization of new isoforms are critical. Even minor variations in amino acid sequences can markedly alter their affinity for ion channels, potentially leading to changes in target specificity [1].

Considering the evolutionary mechanisms that drive the diversity of scorpion venoms and the limited literature on *Tityus obscurus*, a detailed biochemical analysis of its venom is justified. In this study, we conducted a comprehensive proteomic investigation of *Tityus obscurus* venom, with the aim of identifying novel peptides isoforms potentially shaped by evolutionary processes.

## 2. Results

**Figure 1:**
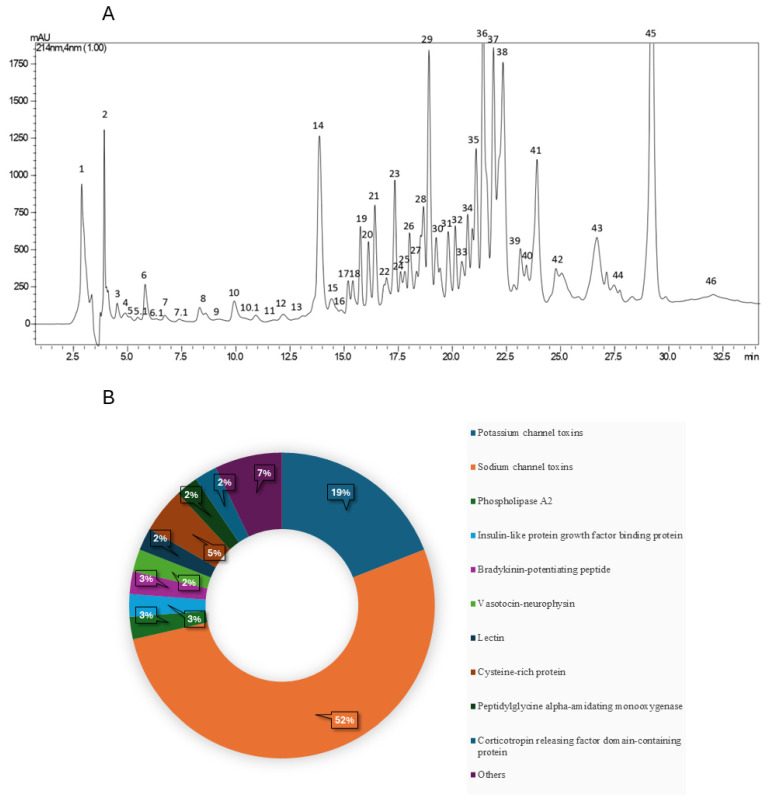
(**A**) Chromatographic profile of *Tityus obscurus* crude venom obtained by C18-RP-HPLC for proteomic analysis focusing on the collection area and respective collected fractions. (**B**) Distribution of the diversity of toxins identified in the venom of the scorpion *Tityus obscurus*; the classifications were made according to the similarity of the peptides and proteins with those already described in the literature.

The chromatographic profile obtained from venom fractionation in the proteomic analysis was divided into 50 distinct fractions (Figure 1A). These fractions were collected using HPLC equipment, lyophilized, enzymatically digested, and subsequently analyzed using mass spectrometry (LC-MS/MS).

In our study, only peptides and proteins with a −10lgP value of 50 or higher were considered for further analysis. Figure 1B shows the molecular diversity identified, categorized based on the number of peptides and proteins found and identified within each group. Additionally, the observed sequence variations were also analyzed and are listed in Table 1.

To further validate our findings, we analyzed the mass spectra of the fragment ions corresponding to each of the proteins listed in Table 2, Table 3 and Table 4. This additional evaluation aimed to enhance the robustness of our results by identifying conserved fragment ions among the peptides, even in the absence of differential regions. Accordingly, peptides exhibiting these conserved fragments are grouped and highlighted in Table 2, Table 3 and Table 4.

Regarding toxins that interact with sodium channels, we identified a total of eight toxins matches and three conserved regions between toxins which were grouped (Table 2). Due to the high similarity among these isoforms, differentiating and classifying the toxins was only possible after prior fractionation, which allowed for their distinction and appropriate grouping. Most of these peptides are currently annotated in UniProt only at the transcriptomic level, which explains their uniform nomenclature (“Potassium channel toxin”). This highlights the pressing need for a comprehensive proteomic update of this venom to refine its molecular characterization, identify novel components, and enhance our understanding of its functional diversity. To streamline the data organization, these peptides were numbered from 1 to 7 in the present work (Table 2).

**Figure 2:**
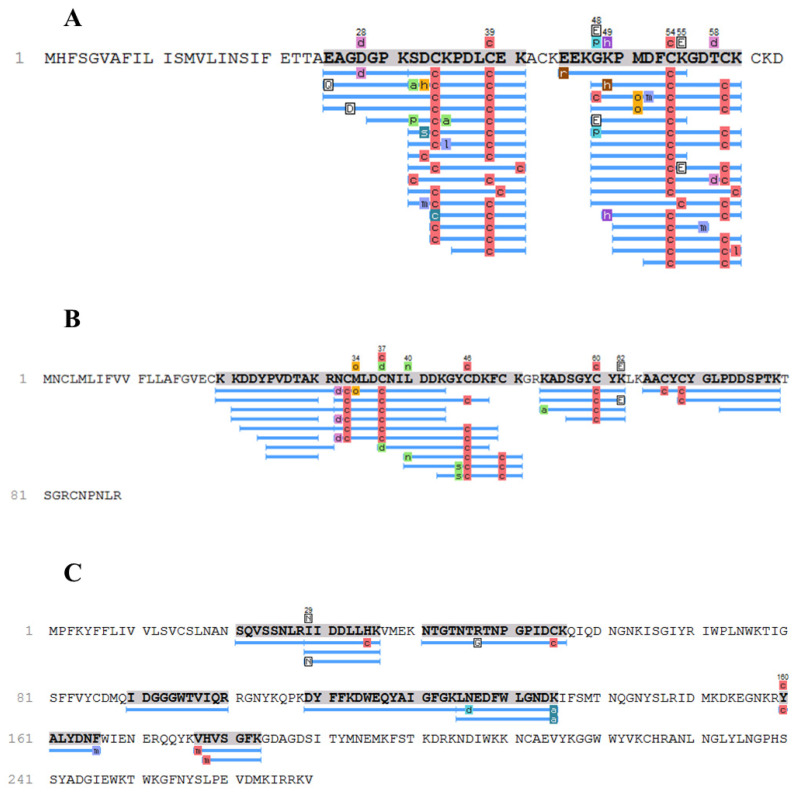
Map coverage representing the three groups of molecules found in the proteomic identification. (**A**) Potassium channel toxin 1 (A0A1E1WVU5); (**B**) Sodium channel toxin 1 (A0A1E1WVY8) and (**C**) Lectin (A0A1E1WVQ4).

**Figure 3:**
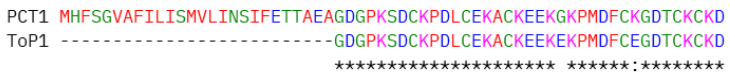
Alignment of Potassium channel toxin 1 (A0A1E1WVU5) and the new isoform identified, named ToP1 by the present work. Data obtained after proteomic analysis of the *Tityus obscurus* venom fractions. Standard Clustal Omega color codes used for amino acid polarity/charge. (PCT1 = Potassium channel toxin 1). * indicates full identity; : denotes a conservative substitution (different amino acids with similar physicochemical properties); a space indicates a non-conservative substitution (amino acids with distinct properties).

Additionally, a novel isoform of a peptide designated as Sodium channel 1 (A0A1E1WVU5) was identified at the proteomic level. This new isoform contains two key amino acid substitutions, the G48E and K55E (Figure 2A and Figure 3), and was named ToP1 by the present work (Table 1).

Regarding toxins that interact with sodium channels, we identified a total of 22 toxins matches and 6 conserved regions between toxins which were grouped (Table 3). We analyzed the peptide fragments for each of the toxins to determine whether all of the listed toxins were differentially identified or shared conserved sequences.

**Figure 4:**
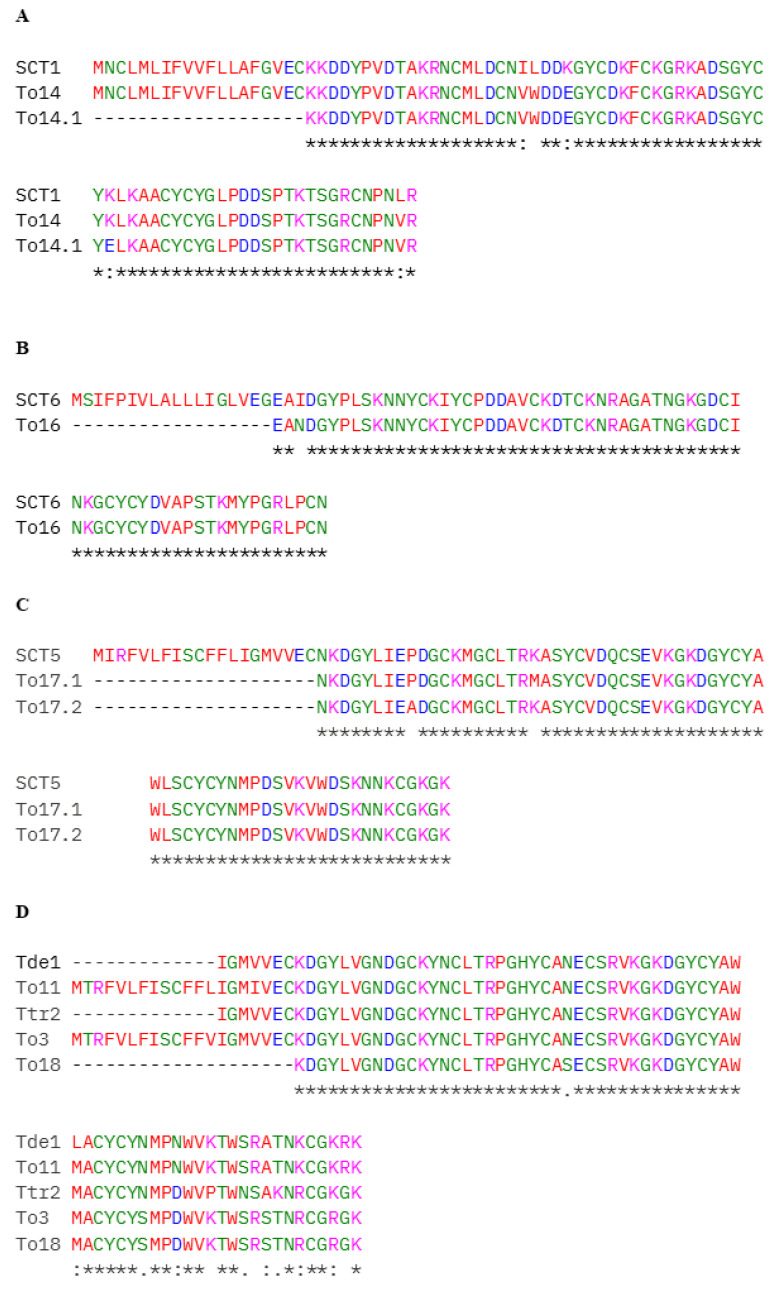

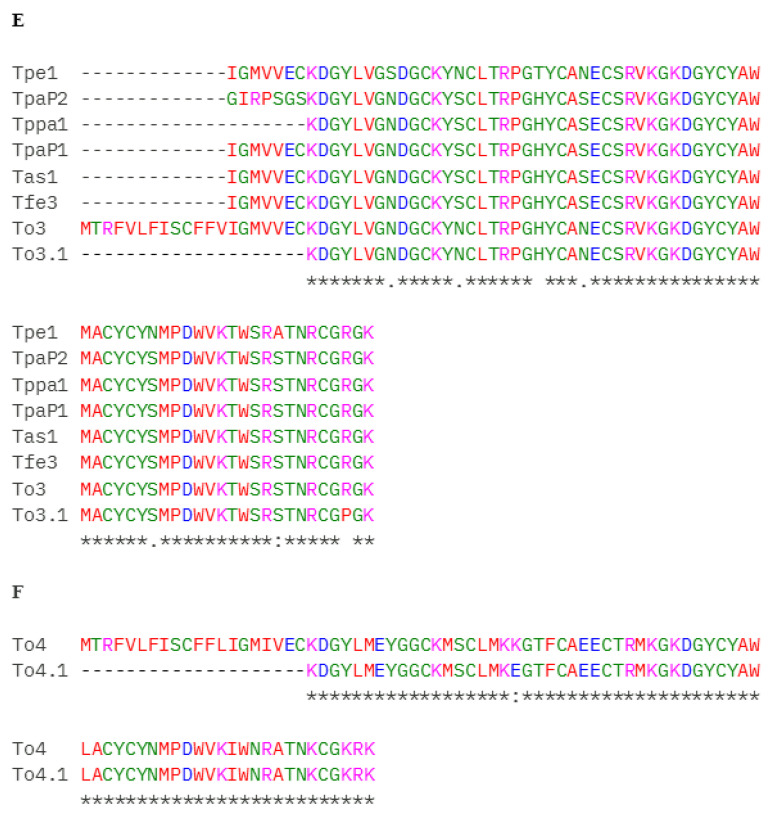
(**A**) Alignment of Sodium channel toxin 1 (A0A1E1WVY8), To14 (H1ZZI3) and the new isoform identified, named To14.1 by the present work. (**B**) Alignment of Sodium channel toxin 6 (A0A1E1WW05) and the new isoform identified, named To16 by the present work. (**C**) Alignment of Sodium channel toxin 5 (A0A1E1WWE3) and the new isoform identified, named To17 by the present work. (**D**) Alignment of Tde1 (A0A7L4XVB3), To11 (H1ZZI0), Ttr2 (A0A7L4XVE5), To3 (P60213) and the new isoform identified, named To18 by the present work. (**E**) Alignment of Tpe1 (A0A7L4XS62), TpaP2 (A0A7L4XT36), Tppa1 (C0HLZ0), TpaP1 (A0A7L4XS46), Tas1 (A0A7L4XS27), Tfe3 (A0A7L4XUN7), To3 (P60213) and the new isoform identified, named To3.1 by the present work. (**F**) Alignment of To4 (P60215) and the new isoform identified, named To4.1 by the present work. Data obtained after proteomic analysis of the *Tityus obscurus* venom fractions. Standard Clustal Omega color codes used for amino acid polarity/charge. (SCT = Sodium channel toxin). * indicates full identity; : denotes a conservative substitution (different amino acids with similar physicochemical properties); . represents a semi-conservative substitution or weak similarity; a space indicates a non-conservative substitution (amino acids with distinct properties).

The same amino acid substitution (K62E) was identified in two peptides previously classified in the literature as Potassium channel toxin 1 (A0A1E1WVY8) and To14 (H1ZZI3), both detected in fraction 28 (Figure 2B and Figure 4A). Sequence alignment confirmed that these peptides are isoforms (Figure 4A) exhibiting high sequence similarity, presenting only four amino acid substitutions. Figure 4A represents the three isoforms of the To14 peptide, including the new isoform identified by the present work, which we designated as To14.1 (Table 1).

In the case of the peptide identified as Sodium channel toxin 6 (A0A1E1WW05), a novel isoform carrying the N21I amino acid substitution was detected (Figure 4B) and named To16. Similarly, for the peptide described in the literature as Sodium channel toxin 5 (A0A1E1WWE3), two novel isoforms were identified, distinguished by the P29A and K40M and P29A amino acid substitution (Figure 4C) in different fractions 36 and 37, respectively. Since this amino acid substitution were found in two distinct fractions (with different elution time), we consider them to be two new isoforms, named To17.1 and To17.2 (Table 1).

Additionally, we also identified the same amino acid substitution (N45S; N32S) in a fragment conserved among four toxins from different species of the genus *Tityus*: toxin To3 (P60213), neurotoxin Ttr2 (A0A7L4XVE5), neurotoxin Tde1 (A0A7L4XVB3), and To11 (H1ZZI0) (Figure 4D). This new isoform was named To18 by the present work (Table 1).

A similar case was observed with R71P, R64P, and R84P in a fragment conserved among seven toxins from different species of the genus *Tityus*: Neurotoxin TpaP2 (A0A7L4XT36), Neurotoxin TpaP1 (A0A7L4XS46), Neurotoxin Tas1 (A0A7L4XS27), Neurotoxin Tfe3 (A0A7L4XUN7), Toxin Tppa1 (C0HLZ0), Neurotoxin Tpe1 (A0A7L4XS62), Toxin To3 (P60213). These amino acid substitutions were found in a conserved fragment among these toxins (Figure 4E), and this new isoform was designated as To3.1 by the present work (Table 1). Furthermore, a novel isoform of the beta-toxin To4 (P60215) was identified and named To4.1 (Table 1), based on the K39E amino acid substitution (Figure 4F).

Beyond ion channel-interacting toxins, several other proteins were identified (Table 4), including phospholipase A2, lectins, and a bradykinin-potentiating peptide, among others. Notably, the most prominent chromatographic peak (labeled as 45 in Figure 1A) was identified as a phospholipase A2 fragment through proteomic analysis.

**Figure 5:**
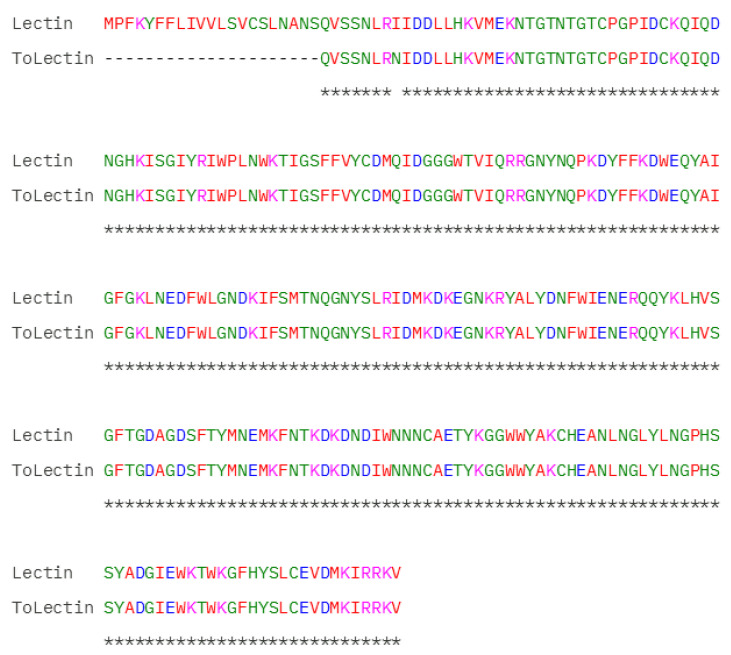
Alignment of Lectin (A0A1E1WVN0) and the new isoform identified, named ToLectin by the present work. Data obtained after proteomic analysis of the *Tityus obscurus* venom fractions. Standard Clustal Omega color codes used for amino acid polarity/charge. * indicates full identity; a space indicates a non-conservative substitution (amino acids with distinct properties).

Among the other molecules in the group that do not belong to the ion channel toxin group, only one substitution was detected: an I29N amino acid substitution in a lectin (Figure 2C and Figure 5). This new isoform was designated as ToLectin (Table 1).

## 3. Discussion

The extensive peptide diversity found in *Tityus obscurus* venom, revealed through chromatographic and mass spectrometry (LC-MS/MS) analyses, reflects the long-term influence of natural selection over millions of years. This evolutionary process has widely influenced the venom of scorpions and other species by changes in diet and predatory behavior. After 400 million years of adaptation, scorpions have developed a venom rich in bioactive molecules, especially peptide toxins, capable of targeting a wide range of molecular structures [22,23]. These variations may lead to differences in toxicity, pharmacological properties, and potential therapeutic applications. Given that venom composition can vary between species, populations, and even individuals, describing and classifying these isoforms provides valuable insights into venom evolution, adaptation, and ecological roles. Moreover, the identification of novel peptide isoforms expands the repertoire of bioactive molecules available for biotechnological and pharmacological research [24], paving the way for the discovery of new drug leads, including peptide-based therapeutics for conditions such as autoimmune diseases, chronic pain, and cancer.

The sample preparation stage is a critical step in proteomic analysis, especially when performing strategic isoform identification. To optimize our proteomic approach, *Tityus obscurus* crude venom was fractionated using reversed-phase high-performance liquid chromatography (C18-RP-HPLC), resulting in the chromatographic profile presented in Figure 1A. This fractionation process was essential for enhancing sample handling during subsequent steps, including reduction, alkylation, trypsinization, and mass spectrometry analysis, thereby ensuring higher precision and reliability in peptide identification

The predominance of toxins targeting K^+^ and Na^+^ channels (approximately 19% and 52%, respectively) in the proteomic analysis was an expected outcome, given that scorpion envenomation is mainly characterized by neurotoxic symptoms. The presence of ion channel-targeting peptides in ancestral scorpions supports their fundamental role in venom function and the evolutionary success of scorpions [5,25].

Our results corroborate previous proteomic studies on *Tityus* scorpions, in which sodium and potassium channel -targeting toxin were also identified: *Tityus stigmurus* [26]; *Tityus obscurus* [3], *Tityus serrulatus* [3], *Tityus discrepans* [27], and *Tityus bahiensis* [1], among others. For Na^+^ channel-targeting toxins, our analysis identified To9 (H1ZZH8), To6 (P84685) and To7 (P84688) toxins classified as a α-toxin [7]. To5 (P84693) was also identified as an α-toxin. Sequence analysis of To5 (P84693) suggests that it may be specific to arthropods [7]. Additionally, the toxin To1 (P60214) was also identified, and it is classified as a β-toxins, while To12 (H1ZZI1), and To15 (H1ZZI4) peptides were also classified as β-toxins To1 (P60214) but presented activity on human NaV1.1–1.6 isoforms [28].

The toxin Sodium channel 6 (A0A1E1WW05) was observed the exchange of amino acids with different chemical characteristics (asparagine for isoleucine). In Sodium Channel 5 (A0A1E1WWE3), we observed the replacement of a polar uncharged residue (proline) with a nonpolar aliphatic residue (alanine), and the substitution of a positively charged residue (lysine) with a nonpolar aliphatic residue (methionine).

Regarding To4 toxin (P60215), the substitution of an amino acid with a positively charged side chain (lysine) for a negatively charged amino acid (glutamic acid/glutamate). The To4 toxin (P60215) is classified as a β-toxin, and it can interact with seven subtypes of sodium channels (hNav1.1–hNav1.7) [20]. The authors also provide data on the specific amino acids responsible for the toxin-channel interactions in each channel subunit. Although the substitutions identified in this study are not mentioned in the aforementioned research, further studies are required to investigate how this may alter To4 binding affinity and function.

Additionally, we identified conserved amino acid substitutions (R71P, R64P, and R84P) in the toxins collectively designated in this study as To3.1. These substitutions were detected across seven isoforms of toxins from different species: Neurotoxin TpaP2 (*Tityus pachyurus*), Neurotoxin TpaP1 (*Tityus pachyurus*), Neurotoxin Tas1 (*Tityus asthenes*), Neurotoxin Tfe3 (*Tityus festae*), Toxin Tppa1 (*Tityus pachyurus*), Neurotoxin Tpe1 (*Tityus perijanensis*), and Toxin To3 (*Tityus obscurus*). Notably, Toxin To3, identified in *Tityus obscurus*, is an α-toxin that interacts with site 3 of vertebrate sodium channels in a voltage-dependent manner [22], where it was originally described as Tc48b. We also detected conserved alterations (N32S; N45S) in a conserved peptide between four toxins: To3 (P60213), neurotoxin Ttr2 (A0A7L4XVE5), neurotoxin Tde1 (A0A7L4XVB3), and To11 (H1ZZI0). Among these toxins is To11 and according to Guerrero-Vargas et al. (2015), this toxin shares sequence similarities with α-toxin and β-toxins. Furthermore, this toxin might present the C-terminal amidated [7].

A novel substitution (K62E) was identified in To14 (H1ZZI3), an α-toxin. New World scorpion toxins, such as To14, are characterized by the presence of a conserved hydrophobic region and the carboxy-terminal stretch has a limited degree of structural freedom. These characteristics make it possible to form several bioactive regions. The exchange of amino acids with different chemical characteristics, of a basic amino acid (K) for an acid amino acid (E), can lead to changes in the chemical characteristics of the toxin. Further studies are needed to show whether and how these changes occur [7].

Among the toxins that act on potassium channels, we identified two alterations (G48E; K55E) in the Potassium channel 1 toxin (A0A1E1WVU5). The first amino acid substitution corresponds to the substitution of an aliphatic amino acid (glycine) for a negatively charged amino acid (glutamate), while the second involves replacing a basic amino acid (lysine) with a negatively charged residue (glutamate).

Among the toxins identified, α-KTx 18.1 (alternative names: Tc32 and To32) is a toxin identified in the venom of *Tityus obscurus* by [3]. This toxin exhibits an inhibitory effect when interacting with Kv1.3 channels of humans T lymphocytes [29]. Notably, Kv1.3 channels have garnered attention as promising therapeutic targets in neuroinflammatory disorders, as their blockade can modulate microglial activation and potentially confer neuroprotection [30].

Beyond ion channel toxins, we identified Phospholipases A2, lectins, bradykinin-enhancing peptides, Insulin-like protein growth factor, Vasotocin-neurophysin, Peptidylglycine alpha-amidating Monooxygenase, and Corticotropin-releasing factor. Phospholipase A2 enzymes play a fundamental role in several biological processes, such as the production of lipid mediators, cell membrane homeostasis, and lipid digestion. Several biological activities have already been described for phospholipases A2 purified from scorpion venom: anti-angiogenic, antitumor, anticoagulant, hemolytic, and neurotoxic activity [31]. Through our analysis, we identified multiple sequence variations presented in Table 1. Among non-toxin proteins, we identified a novel amino acid substitution (I29N) in a lectin molecule (A0A1E1WVN0), involving the substitution of a nonpolar amino acid (isoleucine) with a polar residue (asparagine).

The amino acid substitution analysis is essential, as changes in amino acid composition can have significant functional implications, particularly in modulating toxin-channel interactions. These modifications may represent evolutionary adaptations that enhance venom efficacy. [32] compared the Tb1 toxin from *Tityus bahiensis* with the toxin Ts1 from *Tityus serrulatus*. They demonstrated that the difference in just two amino acid residues between the toxins results in Tb1 being less toxic, as shown by in vitro experiments and supported by the previous literature [32]. Therefore, the amino acid substitution described in this study may alter the toxin’s affinity for specific sodium channels and/or its toxicity [32].

The identification of these novel isoforms is crucial for a deeper understanding, not only of their biochemical properties but also in the context of envenomation. Even minor structural alterations in the peptide can lead to significant changes in the affinity of the toxins for different subtypes of channels [1].

Based on the proteomic analysis, it is noteworthy that enzymes such as metalloproteinases, serine proteases, and hyaluronidases were not found, which was somewhat unexpected, considering that at least one of these enzymes has been identified in proteomic analyses of other species within this genus [1,26,33]. [3], in their transcriptomic analysis of the venom of *Tityus obscurus*, identified transcripts for enzymes like metalloproteinases. However, despite being complementary techniques, transcriptomic data are not always reflected in proteomic analyses. It is also important to note that the cited study did not identify transcripts for phospholipases, which are the molecules found in the predominant peak in the present study. The absence of these enzymes may be directly linked to the evolutionary success of these animals, as their toxins, which target ion channels, appear to be sufficiently effective for their survival. After all, “Nothing in biology makes sense except in the light of evolution” [34].

## 4. Conclusions

In this work, a comprehensive proteomic analysis of *Tityus obscurus* venom was performed. The sample was divided into 50 fractions, leading to identification of 45 proteins, including 8 toxins that interact with K^+^ channels, 22 toxins that interact with Na^+^ channels and 15 other proteins. Additionally, in this analysis, we also identified seven novel isoforms of peptides present in the venom, highlighting the molecular diversity and complexity of this venom.

Venomous and poisonous animals are often described in the literature as arsenals of bioactive molecules, making them valuable subjects for research across multiple scientific disciplines. We report seven new isoforms among peptides that interact with sodium and potassium channels. These amino acid substitutions require further investigation to elucidate their impact on toxin-channel dynamics and the implications of these changes. However, despite these promising findings, certain limitations must be acknowledged. First, while proteomic analysis provided valuable insights, functional validation through electrophysiology or structural studies is necessary to determine how these isoforms influence ion channel activity. Additionally, inter-individual and environmental variability may impact venom composition, suggesting that further studies with larger sample sets are warranted.

Nevertheless, this study significantly expands our understanding of *Tityus obscurus* venom and lays the groundwork for future research into its pharmacological and evolutionary implications. Given the presence of a diverse array of peptides, this venom remains a promising source for discovering novel bioactive compounds with potential therapeutic applications. Furthermore, the creation of comprehensive omics libraries, such as proteomic databases and venom categorizations, is essential for advancing toxicology research. These resources not only facilitate the systematic classification of venom components, but also enhance comparative studies across species, enabling a deeper understanding of venom evolution, functional diversification, and potential biomedical applications.

## 5. Materials and Methods

### 5.1. Reagents and Scorpion Venom

All reagents used in the present study were obtained from Sigma Aldritch© (Darmstadt, Germany) or similar quality, with an analytical purity level of 98%. *Tityus obscurus*’ venom was obtained by electrical stimulation of the telson, from animals collected in the Amazon region, in Santarém and Belterra municipalities in Pará state [2]. The raw lyophilized venom was collected under IBAMA’s license nº 20158-1. A total of 3060 µg of crude venom was used.

### 5.2. RP-HPLC

The lyophilized venom of *Tityus obscurus* was resuspended in 0.1% Trifluoroacetic Acid (TFA) and centrifuged (10,000× *g*) for 10 min, at 4 °C. The supernatant was then analyzed and fractionated by reversed-phase high-performance liquid chromatography (RP-HPLC) in a Shimadzu Prominence binary system (Shimadzu, Kyoto, Japan), coupled to a C18 analytical column (250 × 4.6 mm, 5 μm). UV detection was performed (SPDM 20A, Shimadzu, λ = 214 nm) and separation was achieved by a linear gradient of 10–60% solvent B (90% acetonitrile, containing 0.1% TFA) over A (0.1% TFA) for 30 min at a constant flow of 1 mL·min^−1^. This fractionation approach was implemented to improve the resolution and sensitivity of the proteomic analysis, facilitating the identification and characterization of venom components with higher precision.

### 5.3. Proteomic Analysis

Manually collected fractions (50 µL aliquots) were submitted to in-solution digestion under the following conditions: (1) 5 µL DTT (100 mM dithiothreitol) was added for 30 min at 60 °C; (2) 2.5 µL of iodoacetamide (200 mM) was added for 45 min at room temperature and protected from light; and (3) sample incubation was conducted for at least 12 h at room temperature with 10 µL of trypsin (40 ng/µL in 100 mM ammonium bicarbonate). The reaction was stopped by freezing.

The samples then were analyzed using liquid chromatography mass spectrometry in an ESI-IT-TOF instrument coupled to a UPLC 20A Prominence (Shimadzu, Kyoto, Japan). Samples (50 µL aliquots) were loaded into a C18 column (Kinetex C18, 5 μm; 50 × 2.1 mm) and fractionated by a binary gradient employing as solvents (A) water: acid (999:1) and (B) ACN: water: acid (949:50:1). An elution gradient of 0–40% B was applied for 80 min at a constant flow of 0.2 mL·min^−1^ after initial isocratic elution for 5 min. The eluates were monitored using a Shimadzu SPD-M20A PDA detector before being injected into the mass spectrometer.

The interface was kept at 4.5 kV and 200 °C. The detector operated at 1.95 kV and the argon collision induced fragmentation was set at 55 ‘energy’ value. MS spectra were acquired in positive mode in the 350–1400 *m*/*z* range, and MS/MS spectra were collected in the 50–1950 *m*/*z* range.

Raw LCD Shimadzu data files were converted into Mascot Generic Format (MGF) files using the LCMSolution tool and then loaded into Peaks Studio V7.0 (Bioinformatic Solutions Inc, Waterloo, ON, Canada). Data were processed according to the following parameters: MS and MS/MS error mass were 0.1 Da; methionine oxidation and carbamidomethylation as variable and fixed modification, respectively; trypsin as cleaving enzyme; maximum missed cleavages (3), maximum variable PTMs per peptide (3), and non-specific cleavage (1). Data were analyzed against the “Scorpion” database, compiled in December/2024 by UNIPROT.

## Figures and Tables

**Table 1:** Amino acid substitutions found in the analysis.

Fraction	Description	Access	Amino Acid Substitution	New Isoform Names
14	Potassium channel toxin 1	A0A1E1WVU5	G48E K55E	ToP1
28	Sodium channel toxin 1, Toxin To14-Substitution found in a conserved fragment	A0A1E1WVY8, H1ZZI3	K62E	To14.1
31	Sodium channel toxin 6	A0A1E1WW05	I21N	To16
36/37	Sodium channel toxin 5	A0A1E1WWE3	K40M (Fraction 36) P29A (Fraction 36 and 37)	To17.1 (P29A; K40M)–found in fraction 36 To17.2 (P29A)–found in fraction 37
36/38	Neurotoxin Ttr2, Neurotoxin Tde1, Toxin To3, Toxin To11–Substitution found in a conserved fragment	A0A7L4XVE5, A0A7L4XVB3, P60213, H1ZZI0	N32S N45S	To18
37	Neurotoxin TpaP2, Neurotoxin TpaP1, Neurotoxin Tas1, Neurotoxin Tfe3, Toxin Tppa1, Neurotoxin Tpe1, Toxin To3-Substitution found in a conserved fragment	A0A7L4XT36, A0A7L4XS46, A0A7L4XS27, A0A7L4XUN7, C0HLZ0, A0A7L4XS62, P60213	R71P R64P R84P	To3.1
40	Beta-toxin To4	P60215	K39E	To4.1
44	Lectin	A0A1E1WVN0	I29N	ToLectin

**Table 2:** Toxins that interact with K^+^ channels identified in *Tityus obscurus* venom from proteomic analyses.

Uniprot Code	Fraction	−10lgP	Peptides	Unique Peptides	Nomenclatures
A0A1E1WVU5	9	101.62	4	4	Potassium channel toxin 1
	10	69.91	3	3	
	10.1	96.76	4	4	
	11	66.12	3	3	
	12	57.29	3	3	
	13	87.12	6	6	
	14	136.08	8	7	
	15	63.29	2	2	
	16	81.43	4	4	
	17	50.51	2	2	
	18	53.03	1	1	
	22	89.28	3	3	
	25	54.52	1	1	
P60211	16	55.44	2	2	Potassium channel toxin alpha-KTx 18.1
	17	50.45	2	2	
	18	97.36	2	2	
	25	91.97	2	2	
	26	129.16	4	4	
A0A1E1WVV4	17	52.38	3	3	Potassium channel toxin 2
	34	90.36	7	7	
	35	155.09	10	9	
	37	70.93	3	2	
	38	68.52	4	4	
	40	60.24	2	2	
	42	60.59	4	4	
A0A1E1WVQ0	18	56.64	2	2	Potassium channel toxin 3
	20	54.75	3	3	
P0DQU5	24	122.54	4	4	Potassium channel toxin (*Tityus metuendus*)
A0A1E1WVZ4	24	128.59	4	4	Potassium channel toxin 5
	25	130.52	3	3	
A0A1E1WVV5	27	55.05	2	2	Potassium channel toxin 6
	28	65.77	4	4	
A0A1E1WVU0	44	109.72	6	6	Potassium channel toxin 7
	45	115.22	6	6	
A0A2I9LP49 Q0GY43 A0A1E1WVV4 (Identification of conserved region)	33	97.84	2	2	KTx (*Centruroides hentzi*) Potassium channel toxin TdiKIK (*Tityus discrepans*) Potassium channel toxin 2
	36	103.19	4	4	
P0DQU5 A0A1E1WVP6 (Identification of conserved region)	23	141.08	6	6	Potassium channel toxin (*Tityus metuendus*) Potassium channel toxin 4
A0A1E1WVV4 Q0GY43 (Identification of conserved region)	41	79.15	4	4	Potassium channel toxin 2 Potassium channel toxin TdiKIK (*Tityus discrepans*)

**Table 3:** Toxins that interact with Na^+^ channels identified in *Tityus obscurus* venom from proteomic analyses.

Uniprot Code	Fraction	−10lgP	Peptides	Unique Peptides	Nomenclatures
A0A1E1WVY8	27	181.71	8	2	Sodium channel toxin 1
	28	214.72	16	4	
	29	226.21	23	23	
	30	133.51	6	6	
	32	93.47	4	2	
	33	93.05	4	4	
	45	77.25	3	3	
H1ZZI3	27	171.58	7	1	To14
	28	197.58	13	1	
A0A1E1WW26	32	104.32	5	1	Sodium channel toxin 2
	33	83.85	2	2	
	34	118.95	6	3	
	35	103.35	3	3	
	36	92.28	3	2	
P84685	31	90.79	4	1	Toxin To6
	32	104.61	9	0	
A0A1E1WWK1	30	55.15	3	1	Sodium channel toxin 3
	32	102.86	8	2	
	33	111.43	7	3	
	34	151.33	10	7	
	35	99.58	5	2	
	36	104.82	6	0	
P84688	33	87.55	5	1	Toxin To7
A0A1E1WVS3	31	104.72	5	0	Sodium channel toxin 4
	35	90.77	5	2	
	36	110.45	6	1	
	37	93.43	6	6	
C9X4K8	33	72.30	4	2	Toxin TdNa10 (*Tityus discrepans*)
A0A1E1WWE3	33	51.33	3	3	Sodium channel toxin 5
	35	91.93	4	4	
	36	166.13	13	11	
	37	157.93	9	8	
A0A1E1WW05	28	83.99	3	3	Sodium channel toxin 6
	29	57.99	2	2	
	30	93.69	5	3	
	31	168.89	6	3	
	32	161.52	13	6	
	33	120.15	6	4	
	34	83.96	5	2	
	35	81.58	3	1	
	36	72.73	4	1	
H1ZZI1	37	67.47	2	2	Toxin To12
	38	128.28	9	9	
	39	135.94	9	9	
	40	58.92	3	3	
P60215	39	51.90	1	1	Beta-toxin To4
	40	56.16	2	2	
	41	101.27	5	3	
H1ZZI4	36	146.85	6	6	Toxin To15
	37	169.34	5	5	
	38	152.72	7	7	
	39	154.87	7	7	
A0A1E1WVT2	45	107.00	5	5	Sodium channel toxin 7
P84693	46	64.98	5	5	Toxin To5
P60214	38	129.59	5	1	Beta-mammal/insect toxin To1
	39	139.60	5	1	
	40	91.86	6	3	
P84631	38	126.31	5	1	Tpa2 (*Tityus pachyurus*)
	39	133.87	5	1	
	40	85.63	4	1	
P60213	36	108.69	6	1	Toxin To3
A0A7L4XT36	35	70.32	2	1	Neurotoxin Tpe1 (*Tityus perijanensis*)
	37	91.44	2	2	
	38	54.05	1	1	
A0A7L4XT36	38	109.79	5	3	Neurotoxin TpaP2 (*Tityus pachyuru*)
C0HLM1	32	56.98	3	1	Tma3 (*Tityus macrochirus*)
A0A7L4XVR8	36	57.36	3	0	Neurotoxin Tpe3 (*Tityus perijanensis*)
A0A1E1WVS3 A0A1E1WWK1 (Identification of conserved region)	31	104.72 (A0A1E1WVS3)	5	0	Sodium channel toxin 4 Sodium channel toxin 3
	31	103.89 (A0A1E1WVS3)	5	0	
A0A1E1WVY8 H1ZZI3 (Identification of conserved region)	31	103.88	3	3	Sodium channel toxin 1 To14
	34	77.91	3	3	
	41	75.63	2	2	
	43	67.29	2	2	
P60213 H1ZZI0 A0A7L4XVE5 A0A7L4XVB3 (Identification of conserved region)	35	110.59	5	2	Toxin To3 Toxin To11 Neurotoxin Ttr2 (*Tityus trinitatis*) Neurotoxin Tde1 (*Tityus dedoslargos*)
	37	115.81	4	1	
C9X4K6 H1ZZH8 (Identification of conserved region)	36	117.88	5	5	Toxin TdNa8 (*Tityus discrepans*) Toxin To9
A0A7L4XT36 C0HLZ0 A0A7L4XS46 A0A7L4XS27 A0A7L4XUN7 C0HLZ1 A0A7L4XVD1 Q1I169 A0A7L4XUV3 (Identification of conserved region)	35	101.88	3	1	Neurotoxin TpaP2 (*Tityus pachyurus*) Toxin Tppa1 (*Tityus pachyurus*) Neurotoxin TpaP1 (*Tityus pachyurus*) Neurotoxin Tas1 (*Tityus asthenes*) Neurotoxin Tfe3 (*Tityus festae*) Toxin Tppa2 (*Tityus pachyurus*) Neurotoxin TpaP5 (*Tityus pachyurus*) Toxin Td5 (Fragment) (*Tityus discrepans*) Neurotoxin Tfe2 (*Tityus festae*)
	37	121.07	4	1	
	39	107.73	5	3	
	40	88.60	3	2	
C0HLZ0 A0A7L4XT36 A0A7L4XS46 A0A7L4XS27 A0A7L4XUN7 (Identification of conserved region)	36	101.63	6	1	Toxin Tppa1 (*Tityus pachyurus*) Neurotoxin TpaP2 (*Tityus pachyurus*) Neurotoxin TpaP1 (*Tityus pachyurus*) Neurotoxin Tas1 (*Tityus asthenes*) Neurotoxin Tfe3 (*Tityus festae*)

**Table 4:** Other molecules identified in *Tityus obscurus* venom from proteomic analyses.

Uniprot Code	Fraction	−10lgP	Peptides	Unique Peptides	Nomenclatures
A0A1E1WVX0	7.1	74.09	4	4	Bradykinin-potentiating peptide
	22	65.80	2	2	
	25	61.81	4	4	
A0A1E1WVN4	26	139.09	6	6	Insulin-like protein growth factor binding protein
	27	107.94	5	5	
	30	84.10	3	3	
	31	106.35	4	4	
	32	113.77	5	5	
	33	79.19	4	3	
	34	55.88	2	2	
	41	56.67	1	1	
A0A1E1WVS0	38	81.05	3	3	Vasotocin-neurophysin
	39	98.97	5	4	
	40	99.56	5	5	
	41	100.00	6	6	
C9X4G4	41	73.72	2	2	Uncharacterized protein
	45	53.74	1	1	
A0A1E1WVN8	43	118.06	6	6	Venom protein
A0A1E1WVN0	44	85.20	5	5	Lectin
A0A1E1WVV0	44	99.99	3	3	Cysteine-rich protein 1
	45	82.54	3	3	
	46	65.75	1	1	
A0A1E1WVY9	46	71.48	1	1	Cysteine-rich protein 2
A0A1E1WVQ8	44	91.07	3	3	Peptidylglycine alpha-amidating monooxygenase
A0A1E1WVM2	9	61.08	2	2	Corticotropin-releasing factor domain-containing protein
	44	63.83	1	1	
A0A1E1WWE7	44	77.26	4	4	Phospholipase A2 (Fragment)
	45	87.46	4	4	
A0A1E1WWD3 A0A1D3IY23 (Identification of conserved region)	31	75.56	2	2	Venom toxin Peptide ToAcP
	32	75.71	2	2	
	34	119.70	4	4	
	35	125.48	4	4	
	36	78.92	2	2	
	40	86.38	2	2	
A0A1E1WVR3 A0A1E1WVN2 (Identification of conserved region)	43	66.12	1	1	Secreted protein (Fragment)

## Data Availability

Raw data of mass spectrometry analysis is available at ProteomeXchange, ID JPST003625 PXD061143.
